# Unilateral Upper Cervical Posterior Spinal Cord Infarction after a Neuroendovascular Intervention: A Case Report

**DOI:** 10.1155/2018/5070712

**Published:** 2018-07-05

**Authors:** Kareem Elzamly, Christa Nobleza, Ellen Parker, Rebecca Sugg

**Affiliations:** ^1^Department of Neurology, University of Mississippi Medical Center, Jackson, USA; ^2^Department of Radiology, University of Mississippi Medical Center, Jackson, USA

## Abstract

**Context:**

We describe a case of unilateral posterior upper cervical spinal cord infarction and propose a pathophysiologic mechanism causing this lesion after vertebral artery endovascular intervention.

**Findings:**

A 70-year-old male presented with subacute onset of left hemibody sensory changes and gait instability following a left vertebral angioplasty procedure. MRI cervical spine revealed upper posterior cervical spinal cord infarction (PSCI). After 3 months patient had substantial improvement of his symptoms.

**Conclusion:**

PSCI is rare but can present as a complication from vertebral artery angioplasty procedure. Early diagnosis of PSCI can be achieved with adequate understanding of its clinical signs and the blood supply of the spinal cord.

## 1. Introduction

Spinal cord infarction commonly involves the anterior spinal artery (ASA) territory resulting in motor deficits, sensory disturbances, and urinary incontinence [1, 2]. Posterior spinal cord infarctions (PSCI) are less common, and their diagnosis might be challenging because their presenting symptoms are less characteristic [3]. We describe a rare case of unilateral upper cervical posterior spinal cord infarction following a left vertebral artery (VA) angioplasty and discuss a possible mechanism that lead to this injury.

## 2. Case Report

**Figure 1:**
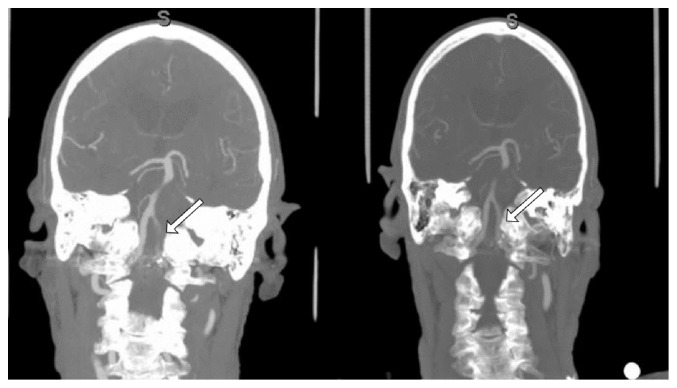
CTA neck coronal MIP planes, (left panel) showing intraluminal thrombus of V4 segment of left vertebral artery (arrow) before cerebral angiogram. Right panel showing patent V4 segment of left vertebral artery (arrow) with resolution of previously seen thrombus.

A 70-year-old male with medical history of hypertension, dyslipidemia, strokes, initially presented to the Neurology clinic with new transient episodes of gait disturbance, left-sided dysmetria, intermittent diplopia, and vertigo. His symptoms were attributed to recurrent transient ischemic attacks involving the posterior circulation. Computed tomography angiogram (CTA) of the neck showed moderate left vertebral artery (VA) V4 and origin stenoses. His symptoms persisted despite being on aspirin, clopidogrel, and atorvastatin. After 4 months, a repeat CTA neck showed progression of left VA stenoses with development of a V4 segment intraluminal thrombus (Figure 1). He was started on oral anticoagulation for 2 weeks without symptoms improvement; therefore, a decision was made to proceed with elective cerebral angiogram with the intent to treat the left VA origin.

Cerebral angiogram one week later showed 90% left VA origin stenosis and distal V4 segment occlusion. Final angiography following left VA angioplasty revealed markedly improved VA flow, and 50% remaining stenosis of the proximal left VA.

**Figure 2:**
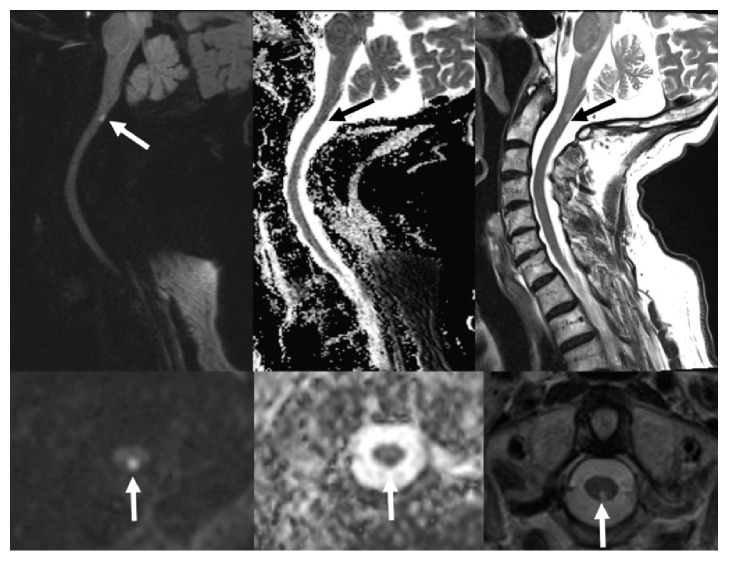
MRI Cervical spine axial and sagittal planes, (left panel) showing hyperintensity on diffusion weighted sequence, hypointensity on ADC sequence (middle panel), and hyperintensity on T2 sequence (right panel) indicating acute spinal cord infarction (arrows).

A few hours postoperatively, the patient developed new onset left upper extremity dysmetria and paresthesia that resolved after 10 minutes. He was discharged from the hospital after 2 days without needing physiotherapy. On the following day, he returned to the emergency room with left hemibody paresthesia and gait unsteadiness. MRI brain showed acute posterior upper cervical cord infarction, confirmed with a dedicated MRI cervical spine (Figure 2). CTA neck showed no restenosis of the left VA origin and patent V4 segment with resolution of previously seen thrombus (Figure 1). His physical examination revealed decreased vibration and proprioception and dysmetria on the left upper and lower extremities, with positive Babinski sign ipsilaterally, and no loss of temperature or pain sensations. He was discharged home the following day with the plan to continue aspirin and clopidogrel and outpatient physiotherapy.

Three months later, the patient reported resolution of left hemibody paresthesia, with mild residual left-hand coordination difficulties and gait imbalance.

## 3. Discussion

Posterior spinal cord infarction is rare [3] and less common than anterior spinal cord infarction (ASCI). This is probably due to the dual posterior spinal arteries (PSA) and the frequent anastomotic networks supplying the dorsal part of the spinal cord. The anterior two-thirds of the spinal cord are supplied by the anterior spinal artery (ASA) which originates from both vertebral arteries at the level of the cervical segment. The ASA also provides blood supply to the central portion of the spinal cord via the sulcal arteries [2, 3].

The paired PSA originate from the vertebral arteries or PICAs and then descend medial to the dorsal roots forming an anastomotic network dorsally that supplies the posterior third of the cord [2, 4]. The ASA and PSA are connected through the pial arterial plexus that supplies the spinal cord periphery [5]. Additionally, the radicular arteries contribute to the blood supply of the spinal cord in different segments after dividing into posterior and anterior branches [4].

The ischemia of vertebrobasilar circulation can present with variety of symptoms such as dizziness, vertigo, headaches, vomit, diplopia, blindness, ataxia, imbalance, and weakness in both sides of the body [6, 7]. Patients with anterior spinal cord ischemia presents with para- or tetraparesis, bladder dysfunction, and bilateral loss of temperature and pain below the level of infarction with preserved vibration and proprioception sensations [2].

In PSCI the dorsal columns, dorsal horns, and posterior portion of lateral columns are affected [8]; however patients can have selective clinical presentations including loss of vibration and proprioception, segmental deep tendon areflexia, and weakness below the level of the lesion [9].

Several causes of PSCIs have been described in previous reports such as syphilitic arteritis [10], cholesterol emboli from atheromatous aortic plaques [11], intrathecal injection of phenol [12], and plasmocytoma [13]. A review of 16 cases of cervical PSCIs showed that the most common causes of cervical PSCI were atherosclerosis and dissection of VA [14].

Few cases of SCIs have been reported as a complication from coronary angiography, resulting in lower extremity weakness and numbness [15, 16]. The proposed mechanism in these previously reported cases were mechanical trauma due to catheter manipulation resulting in plaque rupture with subsequent embolization. Two cases of ASCI after an endovascular coil embolization of basilar tip aneurysm have been described [5]. It was speculated that wedging of a guiding catheter in the VA caused thromboemboli or insufficient perfusion of the radiculomedullary artery and finally spinal cord infarction.

The patient underwent cerebral angiography with improved blood flow in distal VA. However, the remaining proximal segment stenosis might have led to reduction in the flow pressure distally, causing a flow reversal in the VA. We hypothesize that the counteracting stagnant flows of the VA resulted in its gradual occlusion with subsequent hypoperfusion and thromboembolization in the PSA region.

## 4. Conclusion

Posterior cervical spinal cord infarction is a rare complication of VA angioplasty procedure. As symptoms are not characteristic, spinal imaging with diffusion weighted sequences is of great help in making a timely diagnosis.
